# Welcome to volume 8 of *Future Science OA*

**DOI:** 10.2144/fsoa-2021-0124

**Published:** 2021-12-01

**Authors:** Roshaine Wijayatunga

**Affiliations:** 1 Future Science Group, Unitec House, 2 Albert Place, London N3 1QB, UK

Welcome to volume 8 of Future Science OA! In this short article we'd like to give you a quick roundup of the last year's publishing highlights and take a brief look ahead to 2022.

Certainly, 2020 was a unique year given the challenges to the scientific community, and indeed the world as a whole following the COVID pandemic, and 2021 saw the continuation of these challenges along with some attempt to return to a ‘new normal’ after the global vaccination roll-out and an ease of restrictions and lockdown measures. This is a perfect point to say that the completion of volume 7 of Future Science OA would certainly not have been possible without our authors and reviewers, not to mention our internal and external editorial and production teams. We are grateful for all their commitment, cooperation and hard work throughout the course of the last year.

As with all our previous volumes, content published in volume 7 of *Future Science* OA has continued to be of a very high standard, having gone through our rigorous peer-review and revisions process. We have published a variety of article types through the past year, such as shorter opinion-based editorials and commentaries, and longer review and perspective articles, along with original research and case reports.

## Content highlights

Topics in the 2021 volume of *Future Science OA* have been wide ranging and fall into a variety of subject areas, from oncology, immunology, microbiology and infectious diseases, to bioinformatics, genetics and genomics; as well as, urology, gynecology and obstetrics, to name just a few.

We continued to publish interesting and valuable COVID-19-related content in 2021. COVID-19 content performing well on Altmetric included, a perspective paper titled, ‘The COVID-19 pandemic: a threat to antimicrobial resistance containment,’ published in June 2021 and written by Raspail C Founou *et al.* [1] and ‘Cancer patients with COVID-19: a retrospective study of 51 patients in the district of Piacenza, Northern Italy,’ by Luigi Cavanna *et al.*, and published in the January 2021 issue of the journal [2].

Other content featuring as journal highlights on Altmetric include, ‘Use of amantadine in the evaluation of response to chemotherapy in lung cancer: a pilot study,’ authored by Andrew W Maksymiuk *et al.*, published in January 2021 [3], and a research article titled, ‘Antimicrobial and anti-inflammatory activities of commercial aromatizing fragrances?’, by Hagar Bach and Horacio Bach, and published In April 2021 [4].

Some of our top read content for 2021 include a commentary article titled, ‘Announcing the novel class of GABA-A receptor selective positive allosteric modulator antidepressants,’ discussing this novel class of GASPAMA and their mechanism of action [5]. Fasipe *et al.* explain that further research is necessary to ensure the classification of clinically available classes of antidepressants is regularly updated, and that more research is required to determine how, particularly in depressed patients, the interaction of GABAergic, monoaminergic and glutamatergic neurotransmission systems regulate mood and affective disorders [5].

Another well received article, was written by Aravind Akella and Sudheer Akella, titled, ‘Machine learning algorithms for predicting coronary artery disease: efforts toward an open source solution [6]’. In this research article, authors utilised 6 different machine learning algorithms in order to predict presence of coronary artery disease (CAD) in patients within the Cleveland dataset. In short, the results of this study demonstrated that machine learning algorithms can accurately predict for coronary artery disease and research such as this could help in the use of machine learning algorithms as a diagnostic tool for CAD [6].

Further highlights include the research article, ‘Are all wines made from various grape varieties beneficial in the prevention of myocardial infarction and stroke?’, written by Iwaskai *et al.* [7], ‘Inducible clindamycin resistance and *erm* genes in *Staphylococcus aureus* in school children in Kathmandu, Nepal,’ by Timsina *et al.* [8], and an interesting review paper looking into the attributed functions of DHX9 in cancer development and its potential as an antineoplastic target, titled, ‘The enigmatic helicase DHX9 and its association with the hallmarks of cancer,’ by Gulliver *et al.* [9].

## Future Science Future Star Award

This year, the award gained the highest number of applicants since it was launched, along with nearly 10,000 public votes in the final round. The winner of the 2021 Future Science Future Star Award was announced in October – many congratulations to Karolina Pierzynowska, a molecular and cellular neurobiologist from the University of Gdansk (Poland) for taking the win [10]! As with previous winners of the award, Karolina Pierzynowska will now be part of the *Future Science OA* Early Career Advisory Panel and will also be a Mentor for *BioTechniques* LEARN [11].

## Journal statistics

**Figure 1.**
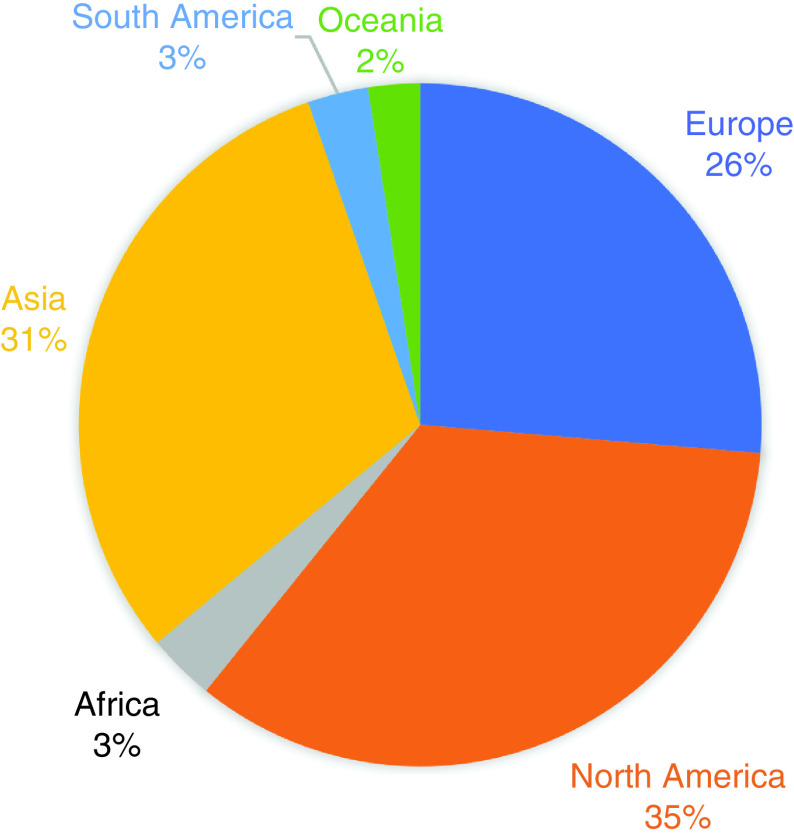
*Future Science OA* readership statistics 2021.

As reflected by the journal readership statistics below, *Future Science OA* has wide reach globally. The readership numbers are very similar to last year, with the majority of readers from Asia, Europe and the Americas (Figure 1).

**Figure 2.**
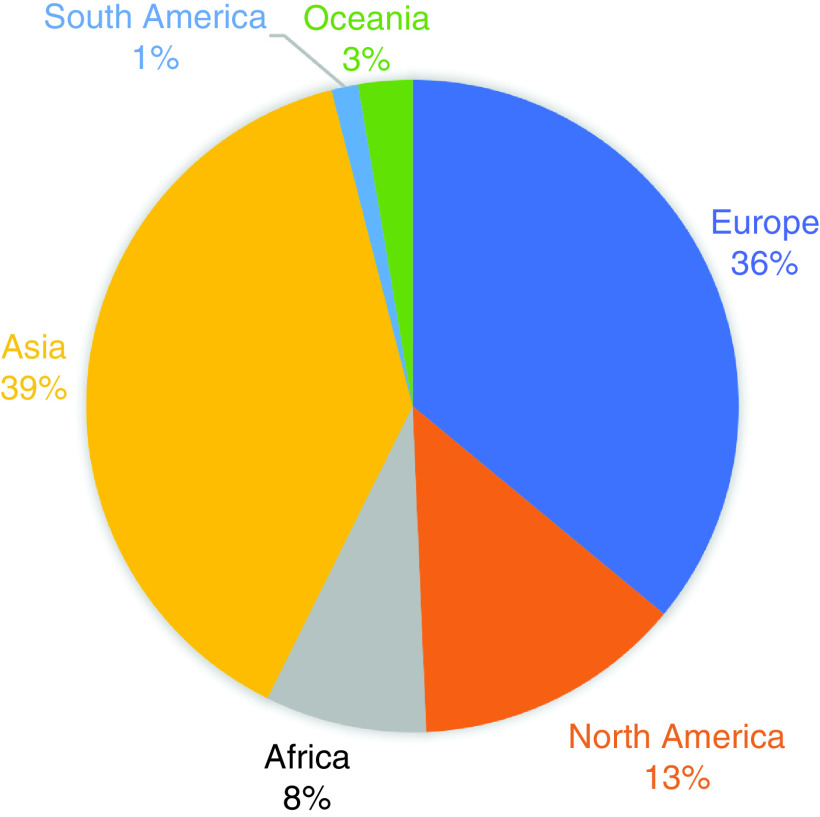
*Future Science OA* authorship statistics 2021.

Journal authorship statistics also show a wide ranging geographic spread as seen in (Figure 2) below, with the majority of authors affiliated to Europe and Asia. Africa, the Americas and Oceania are represented, however, it would be good to increase contributions from these areas in the next year.

We have been able to continue to grant fee-waivers to authors from low-income countries and by publishing all our articles with a Creative Commons CC-BY license we continue to ensure accessibility of our research and content for readers across the globe.

## Looking forward to 2022

This coming year, we would like to continue to build on the success of *Future Science OA* in providing a key resource for researchers within biotechnology, medicine and health, and a platform from which high quality research and content is published, reaching an even wider demographic as previous years. We welcome unsolicited article proposals – please do get in touch if you have any plans for submission or ideas for topic areas we should cover, we would love to hear from you! We also welcome feedback from our readers – please do get in touch!

We believe engaging with the online community via Twitter and other social media channels is very important and if you have not already done so, we would love to welcome you as a follower on Twitter (@fsgfso) [12].

We would like to thank you all again for your interest in and support for *Future Science OA*. We look forward to an even better 2022 and to working with you all this year.

## References

[1] Founou RC, Blocker AJ, Nouborn M The COVID-19 pandemic: a threat to antimicrobial resistance containment. Future Sci. OA 7(8), FSO736 (2021). 34290883 10.2144/fsoa-2021-0012 PMC8204817

[2] Cavanna L, Citterio C, Toscani I Cancer patients with COVID-19: a retrospective study of 51 patients in the district of Piacenza, Northern Italy. Future Sci. OA 7(1), FSO645 (2020). 33432270 10.2144/fsoa-2020-0157 PMC7687531

[3] Maksymiuk AW, Tappia PS, Bux RA Use of amantadine in the evaluation of response to chemotherapy in lung cancer: a pilot study. Future Sci. OA 7(4), FSO679 (2021). 33815824 10.2144/fsoa-2020-0176 PMC8015664

[4] Bach H, Bach H. Antimicrobial and anti-inflammatory activities of commercial aromatizing fragrances. Future Sci. OA 7(6), FSO704 (2021). 34046206 10.2144/fsoa-2020-0194 PMC8147737

[5] Fasipe OJ, Agede OA, Enikuomehin AC. Announcing the novel class of GABA–A receptor selective positive allosteric modulator antidepressants. Future Sci. OA 7(2), FSO654 (2020). 33437518 10.2144/fsoa-2020-0108 PMC7787135

[6] Akella A, Akella S. Machine learning algorithms for predicting coronary artery disease: efforts toward an open source solution. Future Sci. OA 7(6), FSO698 (2021). 34046201 10.2144/fsoa-2020-0206 PMC8147740

[7] Iwasaki M, Murakami M, Ijiri Y, Shimizu M, Yamamoto J. Are all wines made from various grape varieties beneficial in the prevention of myocardial infarction and stroke? Future Sci. OA 7(2), FSO649 (2020). 33437515 10.2144/fsoa-2020-0098 PMC7787155

[8] Timsina R, Shrestha U, Singh A, Timalsina B. Inducible clindamycin resistance and erm genes in Staphylococcus aureus in school children in Kathmandu, Nepal. Future Sci. OA 7(1), FSO361 (2020). 33437500 10.2144/fsoa-2020-0092 PMC7787115

[9] Gulliver C, Hoffmann R, Baillie GS. The enigmatic helicase DHX9 and its association with the hallmarks of cancer. Future Sci. OA 7(2), FSO650 (2020). 33437516 10.2144/fsoa-2020-0140 PMC7787180

[10] https://www.biotechniques.com/news/future-science-future-star-award-2021-and-the-winner-is/?utm_campaign=BioTechniques&utm_content=182790687&utm_medium=social&utm_source=twitter&hss_channel=tw-21155592

[11] https://www.biotechniques.com/about-learn/

[12] https://twitter.com/fsgfso?lang=en

